# Oncogenic signaling upstream of mTORC1 drives lipogenesis and proliferation through SREBP

**DOI:** 10.1186/2049-3002-2-S1-P62

**Published:** 2014-05-28

**Authors:** Stéphane Ricoult, Jessica Yecies, Brendan Manning

**Affiliations:** 1 Genetics and Complex Diseases, Harvard School of Public Health, Boston, MA, USA; 2 Genentech, South San Francisco, CA, USA

## Background

The mechanistic target of rapamycin (mTOR) is a central regulator of cell growth and proliferation, and its aberrant activation is frequent in cancer [1]. We have previously shown that sterol regulatory element binding protein (SREBP) is a major transcriptional effector of mTOR complex 1 (mTORC1) signaling [2]. SREBP is a transcription factor that stimulates the expression of genes involved in the *de novo* synthesis of lipids [3]. Since mTORC1 is commonly activated in cancer through upstream oncogenic signaling pathways and enhanced lipogenesis is a metabolic hallmark of tumor cells, we hypothesize that the mTORC1-SREBP pathway is important for tumor metabolism and growth.

## Materials and methods

We used a panel of breast cancer cell lines with various mutations resulting in constitutive mTORC1 activation, as well as MCF10a cells transformed with oncogenic KRAS or PIK3CA to explore the role of SREBP in mTORC1-driven cancers. We used chemical inhibitors and siRNAs to assess the dependence of our breast cancer systems on the mTORC1-SREBP pathway for *de novo* lipogenesis, proliferation, growth, and cell survival.

## Results

Using both the panel of breast cancer cell lines and the isogenic MCF10a cell lines, we have revealed an essential role for SREBP in driving lipogenesis and proliferation in tumor cells. Our results indicate that SREBP and its lipogenic targets are activated in cell lines with oncogene-induced mTORC1 signaling, and this activation was ablated by mTOR inhibitors. mTORC1 signaling promotes *de novo* lipogenesis in an SREBP-dependent manner in cancer cells. In addition, we found that the mTORC1-stimulated activation of SREBP2 promotes cell growth, proliferation, and survival of breast cancer cells. We are in the process of identifying the essential functions of SREBP2 in breast cancer and assessing the dependence of breast cancer cells on SREBP2 *in vivo*. These findings emphasize the therapeutic potential of targeting lipid metabolism in cancers, particularly those with activated mTORC1 signaling.
